# Understanding Dietary Habit Changes during the COVID-19 Pandemic in Italy

**DOI:** 10.3390/nu14040850

**Published:** 2022-02-18

**Authors:** Giuseppe Grosso

**Affiliations:** Department of Biomedical and Biotechnological Sciences, University of Catania, 95123 Catania, Italy; giuseppe.grosso@unict.it

A recently published study by Cicero et al. [1] gained a great deal of attention and was among the most cited articles published in *Nutrients*. The study investigated for the first time the changes in dietary habits in older individuals in quarantine living in an Italian northern rural area.

The COVID-19 pandemic is testing people’s resilience to rapidly adapt to global recommendations, guidelines, or even governmental mandatory restrictions to their usual lifestyle behaviors, customs and routines, including dietary habits. As return to normality is still far from happening, people are now facing a “new normality” in which old and new routines coexist with the presence of the COVID-19. However, the past mandatory lockdown period, the current remote working from home, the limits in retail shops’ numbers of customers, and the general attention to health-conscious behaviors keeping people safe from exposure to the virus and limiting the risk of infection are inevitably shaping a new (possibly momentary) era in which people are spending much longer time at home. From an epidemiological point of view, these new habits may substantially affect the physical and mental health of individuals, resulting in a demanding need for research studies documenting such changes and providing supportive data for potential interventions.

There is convincing evidence that obesity plays a role in risk of infection, severity of symptoms, probability of hospitalization, and even risk of mortality [2]. However, changes in physical activity and dietary habits due to the pandemic are now hypothesized to be independent contributing factors to potentially influence the COVID-19 pandemic [3]. Among regions with the highest impact of COVID-19 infections, Italy has been among the first in Europe to face the pandemic and react with restrictions without previous experience from other countries. It is still unclear whether dietary factors may have played a role in the post-lockdown era and whether these trends are continuing or rather stopped. Previous studies exploring the dietary habits during the lockdown period in Italian individuals showed substantially mixed results. Some studies observed an increased intake of unhealthy foods and worsening of adherence to the Mediterranean diet [4,5,6]. Other studies reported that the first Italian lockdown led to a higher intake of foods characterizing the Mediterranean dietary pattern [7], with a significant increase in vegetables, whole grains and water consumption, suggesting an improvement in dietary quality [8]. A collective online survey for the Italian population showed mixed findings, with part of participants worsening their dietary habits and part improving [9]. Similarly, another study showed that about one-third of respondents participating in a survey conducted in North Italy reported a worsening of eating habits while a smaller proportion of individuals improved their daily habits [10]. The mixed results obtained in the aforementioned studies may be explained by an interesting observation from Grant and colleagues [11] suggesting that during the lockdown, individuals with healthy dietary habits tended to keep or even improve their behaviors while those with unhealthy ones were more likely to worsen their dietary choices.

With the pandemic continuing to affect people’s lives, similar questions arise concerning dietary habits, such as whether they are affected during the quarantine periods after being tested positive for COVID-19. The study of Cicero et al. [1] provided for the first time evidence of changes in dietary habits in older individuals in quarantine living in an Italian northern rural area, characterized by a significant worsening of diet quality and substantial increase in general food intake. Although this data is not representative for the whole Italian population and too preliminary to draft conclusions from, it may be hypothesized that we may expect similar trends observed as for the lockdown period, with people likely to exacerbate their previous dietary habits resulting in a potential threat for less resilient and more fragile individuals, patients with pre-existing health conditions (especially metabolic disorders including obesity), and socially disadvantaged ones with no access to healthier food products. Further studies are needed to better investigate this hypothesis while public authorities and general practitioners might consider that the quarantine period could worsen health disparities among citizens and intervene accordingly on patients at higher risk.
